# Neuroprotective effects of 1‐O‐hexyl‐2,3,5‐trimethylhydroquinone on ischaemia/reperfusion‐induced neuronal injury by activating the Nrf2/HO‐1 pathway

**DOI:** 10.1111/jcmm.15659

**Published:** 2020-07-17

**Authors:** Chaoliang Tang, Yida Hu, Haiyan Lyu, Jie Gao, Jiazhen Jiang, Xiude Qin, Yuanbo Wu, Jiawu Wang, Xiaoqing Chai

**Affiliations:** ^1^ Department of Anesthesiology, The First Affiliated Hospital of USTC, Division of Life Sciences and Medicine University of Science and Technology of China Hefei China; ^2^ Department of Anesthesiology Renmin Hospital of Wuhan University Wuhan China; ^3^ Department of Neurology, Shanghai General Hospital Shanghai Jiao Tong University Shanghai China; ^4^ Department of Anesthesia, Critical Care & Pain Medicine, Massachusetts General Hospital Harvard Medical School Boston MA USA; ^5^ Department of Emergency, Huashan Hospital North Fudan University Shanghai China; ^6^ Department of Neurology, Shenzhen Traditional Chinese Medicine Hospital The Fourth Clinical Medical College of Guangzhou University of Chinese Medicine Shenzhen China; ^7^ Department of Neurology, The First Affiliated Hospital of USTC, Division of Life Sciences and Medicine University of Science and Technology of China Hefei China

**Keywords:** 1‐O‐Hexyl‐2,3,5‐trimethylhydroquinone, cerebral ischaemic/reperfusion, HO‐1, Nrf2, PC12 cells

## Abstract

1‐O‐Hexyl‐2,3,5‐trimethylhydroquinone (HTHQ), a lipophilic phenolic agent, has an antioxidant activity and reactive oxygen species (ROS) scavenging property. However, the role of HTHQ on cerebral ischaemic/reperfusion (I/R) injury and the underlying mechanisms remain poorly understood. In the present study, we demonstrated that HTHQ treatment ameliorated cerebral I/R injury in vivo, as demonstrated by the decreased infarct volume ration, neurological deficits, oxidative stress and neuronal apoptosis. HTHQ treatment increased the levels of nuclear factor erythroid 2–related factor 2 (Nrf2) and its downstream antioxidant protein, haeme oxygenase‐1 (HO‐1). In addition, HTHQ treatment decreases oxidative stress and neuronal apoptosis of PC12 cells following hypoxia and reperfusion (H/R) in vitro. Moreover, we provided evidence that PC12 cells were more vulnerable to H/R‐induced oxidative stress after si‐Nrf2 transfection, and the HTHQ‐mediated protection was lost in PC12 cells transfected with siNrf2. In conclusion, these results suggested that HTHQ possesses neuroprotective effects against oxidative stress and apoptosis after cerebral I/R injury via activation of the Nrf2/HO‐1 pathway.

## 1 INTRODUCTION

With the acceleration of an ageing population and formation of unhealthy living habits, the incidence of stroke is increasing in the world yearly. Notably, as a common neurological disease, stroke remains the second leading cause of death worldwide, and the second most common cause of disability adjusted life‐years, yet its disease burden has been under‐recognized.^1^, ^2^ Clinical data showed that ischaemic strokes account for over 80% of all stroke cases.^3^ Most cases of ischaemic stroke are due to the narrowing or occlusion of the cerebral arteries and insufficiency of cerebral blood supply, which can lead to nerve injury and neurological deficits. The early intervention strategy of ischaemic stroke is to restore blood supply in the infarct and ischaemic region. However, the reperfusion process may further aggravate ischaemia‐induced cerebral injury, which known as cerebral ischaemic/reperfusion (I/R) injury.^4^, ^5^, ^6^

Accumulating evidence indicates that oxidative stress plays a pivotal role in cerebral I/R injury. Oxidative stress is involved in inflammation and neuronal apoptosis and is also consequences of cerebral I/R injury, and this process, in turn, aggravates brain injury after stroke.^7^, ^8^ Thus, great attention has been addressed to the identification of novel pharmacological strategies able to mitigate the negative effects of oxidative stress in stroke. Nuclear factor E2–related factor 2 (Nrf2), a basic leucine zipper transcription factor, is one of the master regulators of defence against endogenous oxidations. Under conditions of oxidative stress, Nrf2 binds to the antioxidant response element (ARE) sequences and promotes the expression of antioxidant enzymes, including haemoxygenase‐1 (HO‐1).^9^, ^10^, ^11^ Indeed, data from numerous studies indicate that Nrf2 plays an important role in the protection of brain from I/R injury.^12^, ^13^ Satoh T et al reported that enhancive Nrf2 activity exerts a neuroprotective mechanism following cerebral I/R injury.^14^

1‐O‐Hexyl‐2,3,5‐trimethylhydroquinone (HTHQ), a lipophilic phenolic agent, has potent antioxidant activity and reactive oxygen species (ROS) scavenging properties.^15^ Jung YR et al reported that HTHQ could attenuate dimethylnitrosamine‐induced liver fibrosis by reduction of lipid peroxidation and anti‐fibrogenic effects.^16^ The recent research indicates that HTHQ can ameliorate dopaminergic neuronal cell death induced by oxidative stress in vitro.^17^ However, the role of HTHQ on cerebral I/R injury and the underlying mechanisms remains poorly understood. In the present study, we found that HTHQ significantly ameliorates cerebral I/R injury in vivo and protects PC12 cells against hypoxia and reperfusion (H/R) injury in vitro via Nrf2‐mediated antioxidant pathway.

## 2 MATERIALS AND METHODS

### 2.1 Reagents

1‐O‐Hexyl‐2,3,5‐trimethylhydroquinone was purchased from MedChemExpress LLC. Terminal deoxynucleotidyl transferase‐mediated dNTP nick end labelling (TUNEL) assay kit was purchased from Roche Company (Basel, Switzerland). Superoxide dismutase 1 (SOD), catalase (CAT), malondialdehyde (MDA) and glutathione (GSH) assay kits were purchased from Nanjing Jiancheng Bioengineering Institute. ROS assay kit was purchased from Thermo Fisher Scientific. Annexin V‐APC/7‐AAD apoptosis detection kit was purchased from BD Biosciences. A nuclear‐cytosol extraction kit was purchased from Applygen Technologies Inc. Bicinchoninic acid (BCA) protein assay kits were purchased from Thermo Fisher Scientific Inc. Antibodies for Nrf2 (1:1000 dilution), HO‐1 (1:1000 dilution) and β‐actin (1:1000 dilution) were purchased from Abcam. The secondary antibodies and goat anti‐rabbit IgG were obtained from LI‐COR Biosciences. All other chemicals were of analytical grade.

### 2.2 Animals and treatment

Animal care and procedures were conducted according to the National Institutes of Health Guide for the Care and Use of Laboratory Animals and approved by the Animal Care and Use Committee of Renmin Hospital of Wuhan University.

Male C57BL/6J mice weighing 20‐25 g purchased from Beijing Vital River Laboratory Animal Technology Co., Ltd.. All mice were housed with a well‐ventilated environment (22‐24°C; humidity, 55‐65%) and maintained in 12‐hour light‐dark cycle for at least 1 week prior to experimentation. The animals had free access to standard rodent diet and water.

### 2.3 Mouse models of transient focal cerebral ischaemia and drug treatment

Focal cerebral ischaemia was induced by middle cerebral artery occlusion (MCAO) in mice using the intraluminal filament technique as described previously.^18^ Briefly, the animals were anaesthetized and then placed in a supine position and located in a warm surrounding (32‐36°C). Next, a midline incision was created, and skin and muscle were separated bluntly. Then, the right common and external carotid arteries were isolated and ligated, and the internal carotid artery was distally clamped with a microvascular clip. A 6‐0 nylon suture with rounded tip was inserted via a small incision into the common carotid artery and advanced to the carotid bifurcation to occlude the opening of the middle cerebral artery. After ischaemia for 30 minutes, the suture was withdrawn to allow reperfusion. As for sham‐operated mice, the right common and external carotid arteries were isolated, but not inserted suture.

The mice were randomly assigned into four groups: Sham, MCAO, HTHQ 100 and HTHQ 200 group. The sham and MCAO groups received an equivalent volume of Tween‐80 (vehicle) for three consecutive days. The HTHQ 100 and HTHQ 200 groups were orally administered with HTHQ (100 or 200 mg/kg) for three consecutive days before MCAO. The operation was performed after 30 minutes the last administration.

### 2.4 Evaluation of neurological deficits

After 24 hours of MCAO, an investigator who blind to the experimental protocol assessed the mice using Longa et al described neurologic scoring system.^19^ The scoring criteria are the following: 0, no neurological deficits; 1, inability to fully extend the contralateral forepaw; 2, circling to paretic side; 3, inability to contralateral side; and 4, inability to walk spontaneously and lack of response to stimulation.

### 2.5 Triphenyl tetrazolium chloride staining

After neurological evaluation, the mice were killed and the whole brain was rapidly collected for triphenyl tetrazolium chloride (TTC) staining. Briefly, the brain was cooled in iced saline for 10 minutes and sliced into 2‐mm‐thick coronal sections. The sections were immersed in 2% TTC at 37°C 10‐15 minutes in the dark and then transferred to 10% buffered formalin solution. The brain slices were photographed using a digital camera, and infarct size was measured using ImageJ. The infarct volume for each brain was calculated using the following formula: infarct volume ratio (%) = total infarct volume/ total volume of brain × 100%.

### 2.6 Brain water content

The mice were killed 24 hours after MCAO, and the brains were carefully removed. The wet weight was measured promptly by weighing the ischaemic hemispheres. The dry weight was measured by weighing the samples dried at 105°C for 24 hours. The brain water content was calculated using the following formula: Brain water content (%) = (wet weight − dry weight)/wet weight × 100%.

### 2.7 Oxidative stress detection

At the end of the experiment, the brain tissues were immediately removed and washed with prechilled saline, and then dried on filter paper. The brain tissues were prepared as 10% tissue homogenate in a homogenizer and centrifuged at 3000 rpm for 10 minutes to collect the supernatant. As for the cell, PC12 cells were lysed using 0.2% Triton X‐100 and collect the supernatant. SOD, CAT, GSH activities and MDA contents in the brain tissues were measured by commercially available kits. All measure operation steps were strictly followed the manufacturer's instructions.

### 2.8 TUNEL assay

As soon as the mice were killed, the brain tissues were immediately removed and fixed with 4% paraformaldehyde for 24 hours. Then, the tissues were embedded in paraffin and sectioned at a thickness of 4‐5 μm. The slices were stained with TUNEL reagents and DAPI solution according to the manufacturer instructions. Cells were separately counted in five fields randomly chosen under a microscope at 40× magnification. The number and percentage of positive cells in the region were calculated to evaluate the extent of cell apoptosis.

### 2.9 Western blot

The ischaemic side cerebral cortex was collected and lysed using RIPA buffer and centrifuged at 13 523 *g* at 4°C for 30 minutes to obtain total protein. The nuclear and cytosol fractions were isolated using a nuclear‐cytosol extraction kit. Protein concentrations were measured using a BCA protein assay kit and then fractionated on SDS‐PAGE gels. The protein was transferred onto Immobilon NC membranes and blocked with 5% non‐fat milk in Tris‐buffered saline/Tween‐20 at room temperature for 2 hours. Then, the membranes were incubated with different primary antibodies at 4°C overnight and appropriate secondary antibodies at room temperature for 2 hours. The protein blots were scanned and quantified using a two‐colour infrared imaging system (Odyssey; LI‐COR). The intensity of the blots was normalized to that of β‐actin.

### 2.10 Cell culture and genetic manipulation

The PC‐12 cells were purchased from the China Centre for Type Culture Collection. The cells were cultured in DMEM medium (Hyclone Laboratories) supplemented with 10% FBS and 100 units/mL penicillin/streptomycin at 37°C under 5% CO_2_ and 100% humidity. To knockdown the genes, si‐Nrf2 were designed and manufactured according to the previous literature. Briefly, the PC‐12 cells were transfected using Lipofectamine 2000 (Thermo Fisher Scientific) according to the protocol recommended.

### 2.11 Hypoxia and reperfusion (H/R) injury and drug treatment

For hypoxic treatment, the PC‐12 cells were cultured in preconditioned hypoxic medium under 5% CO_2_ and 95% N_2_ in a humidified chamber (Binder, CB‐210 hypoxia workstation) for 6 hours. Following hypoxia injury, the hypoxic medium was replaced with fresh medium and the cells were cultured under normal growth conditions 18 hours of reoxygenation. Meanwhile, the cells were exposed to HTHQ for 3 hours before H/R injury.

### 2.12 Cell apoptosis

After H/R injury, the cell apoptosis was detected by Annexin V‐APC/7‐AAD apoptosis detection kit. Briefly, PC12 cells were collected and washed twice with PBS and resuspended with 500 μL binding buffer suspension. After that, the cells were incubated with 5 μL Annexin V‐APC and 7‐AAD at 37°C for 15 minutes in dark. Flow cytometer (BD Biosciences) was used to detect the percentage of apoptotic cells, and FlowJo software was used for flow cytometry analysis.

### 2.13 Statistical analysis

SPSS 21.0 software (SPSS) was used for statistical analysis. The data are expressed as the means ± standard deviation (SD). Statistical comparisons among multiple groups were carried out using one‐way analysis of variance (ANOVA) tests. Least significant difference t‐tests were used to make intergroup comparisons. *P* < .05 was considered statistically significant.

## 3 RESULTS

### 3.1 HTHQ treatment ameliorates cerebral I/R injury in vivo

**FIGURE 1:**
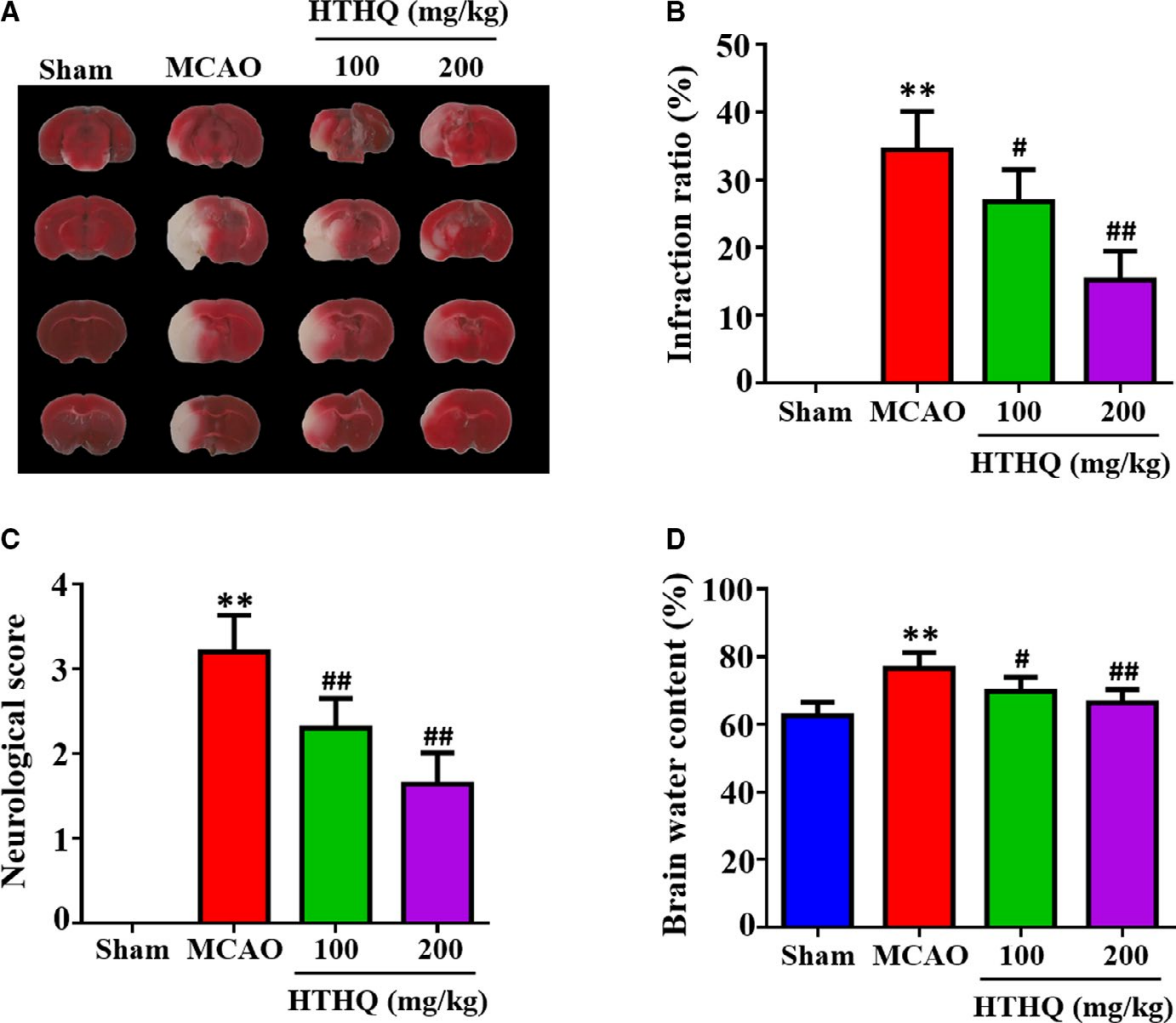
HTHQ treatment ameliorates cerebral I/R injury in vivo. A, Representative brain sections of TTC staining. Pale areas represent infarcted brain. B, Quantification results of infarct volume ration (n = 8). C, Quantification results of neurological deficit scores (n = 8). D, Quantification results of brain water content (n = 8). **P < .01 vs the Sham group; ^#^ *P* < .05 and ^##^ *P* < .01 vs the MCAO group

To investigate the potential role of HTHQ in cerebral I/R injury, we evaluated the infarct volume ratio, neurologic scores and brain water content in mice. TTC staining and neurological function scores demonstrated that the infarct volume ratio and neurologic scores in the MCAO group were obviously increased compared with that in the Sham group, whereas HTHQ significantly decreased the infarct volume ratio and neurologic scores dose dependently (Figure 1A‐C). Moreover, HTHQ treatment significantly ameliorated brain water content at the two doses investigated (Figure 1D).

### 3.2 HTHQ treatment attenuates oxidative stress following cerebral I/R

**FIGURE 2:**
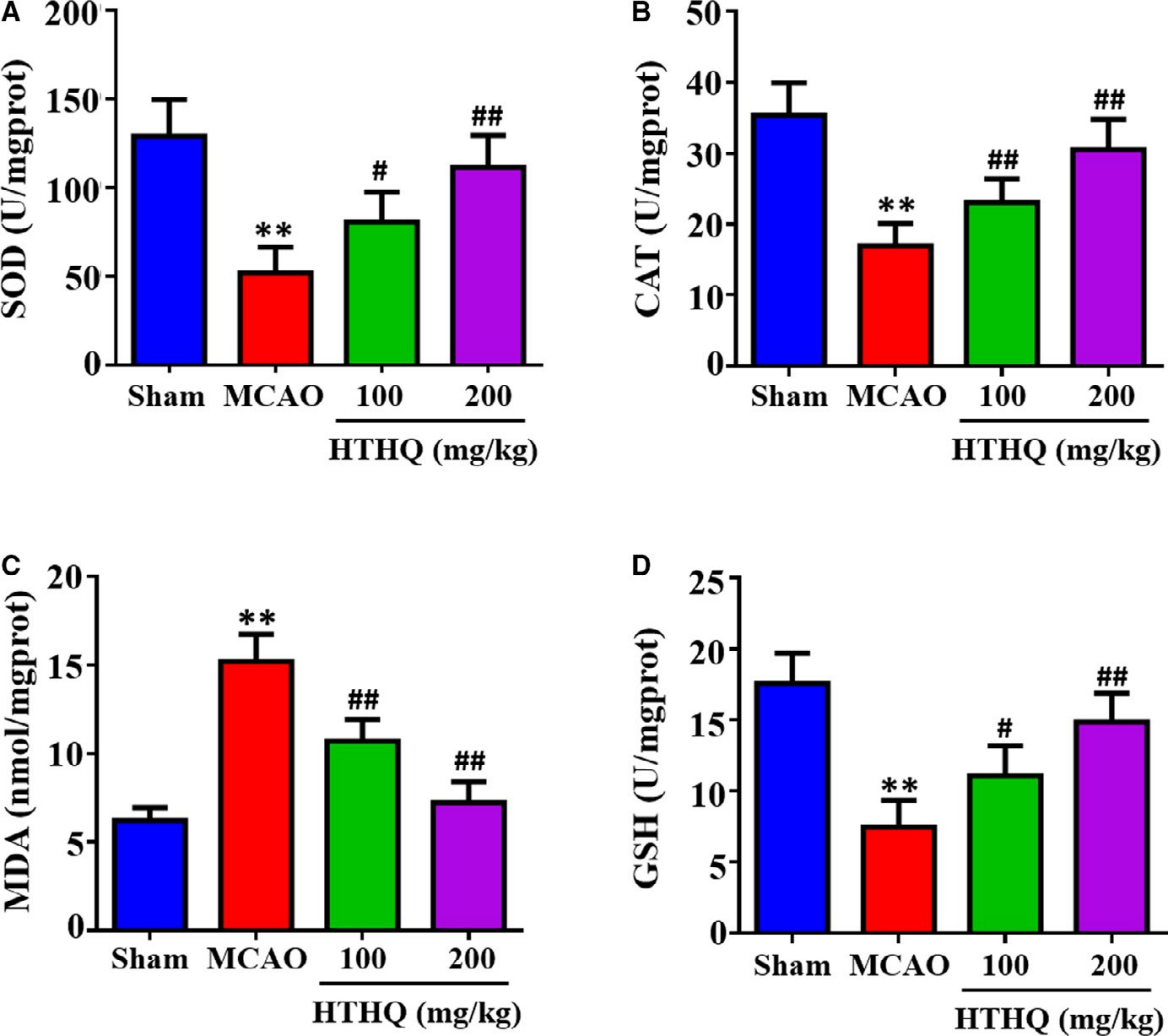
HTHQ treatment attenuates oxidative stress following cerebral I/R. The levels of superoxide dismutase (SOD) (A), catalase (CAT) (B), malondialdehyde (MDA) (C) and glutathione (GSH) (D) in the brain of mice (n = 6). **P < .01 vs the Sham group; ^#^ *P* < .05 and ^##^ *P* < .01 vs the MCAO group

To assess the effects of HTHQ on oxidative stress following cerebral I/R, we measured the SOD, CAT, GSH activities and MDA contents in the brain tissues. The results revealed that the SOD, CAT and GSH activities in the MCAO group were significantly lower and MDA contents were significantly higher than those in the Sham group (Figure 2A‐D). However, HTHQ treatment significantly restored SOD, CAT and GSH activities and decreased MDA contents compared with the MCAO group (Figure 2A‐D). These data indicated that HTHQ treatment attenuates oxidative stress following cerebral I/R.

### 3.3 HTHQ treatment decreases neuronal apoptosis following cerebral I/R

**FIGURE 3:**
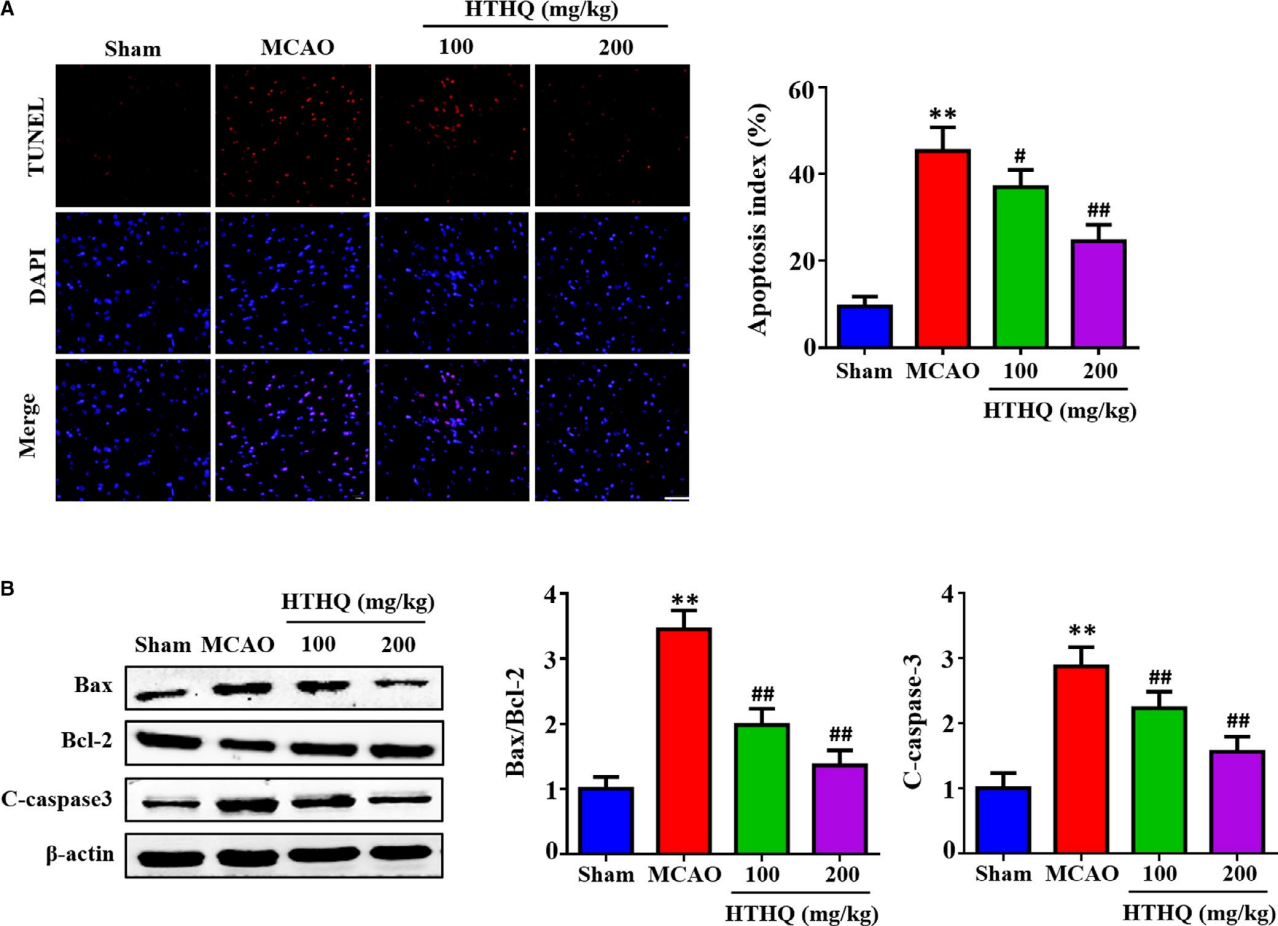
HTHQ treatment decreases neuronal apoptosis following cerebral I/R (A) Images and quantifications of apoptotic cell in the ischaemic cortex of mice (n = 5). (B) Western blots showing the level of Bax, Bcl‐2 and cleaved caspase‐3 in mice following cerebral I/R (n = 4). **P < .01 vs the Sham group; ^#^ *P* < .05 and ^##^ *P* < .01 vs the MCAO group

To further assess the neuroprotective effects of HTHQ, we used the TUNEL staining to measure the neuronal apoptosis. The results demonstrated that the apoptotic index in the MCAO group was obviously increased compared with that in the Sham group, whereas HTHQ treatment significantly decreased neuronal apoptosis following I/R (Figure 3A). In addition, the levels of Bax/Bcl‐2 and cleaved caspase‐3 in the MCAO group were significantly higher than those in the Sham group (Figure 3B). However, HTHQ treatment significantly attenuated the increased Bax/Bcl‐2 and cleaved caspase‐3 levels after MCAO treatment (Figure 3B).

### 3.4 HTHQ against cerebral I/R injury via Nrf2 antioxidant pathway

**FIGURE 4:**
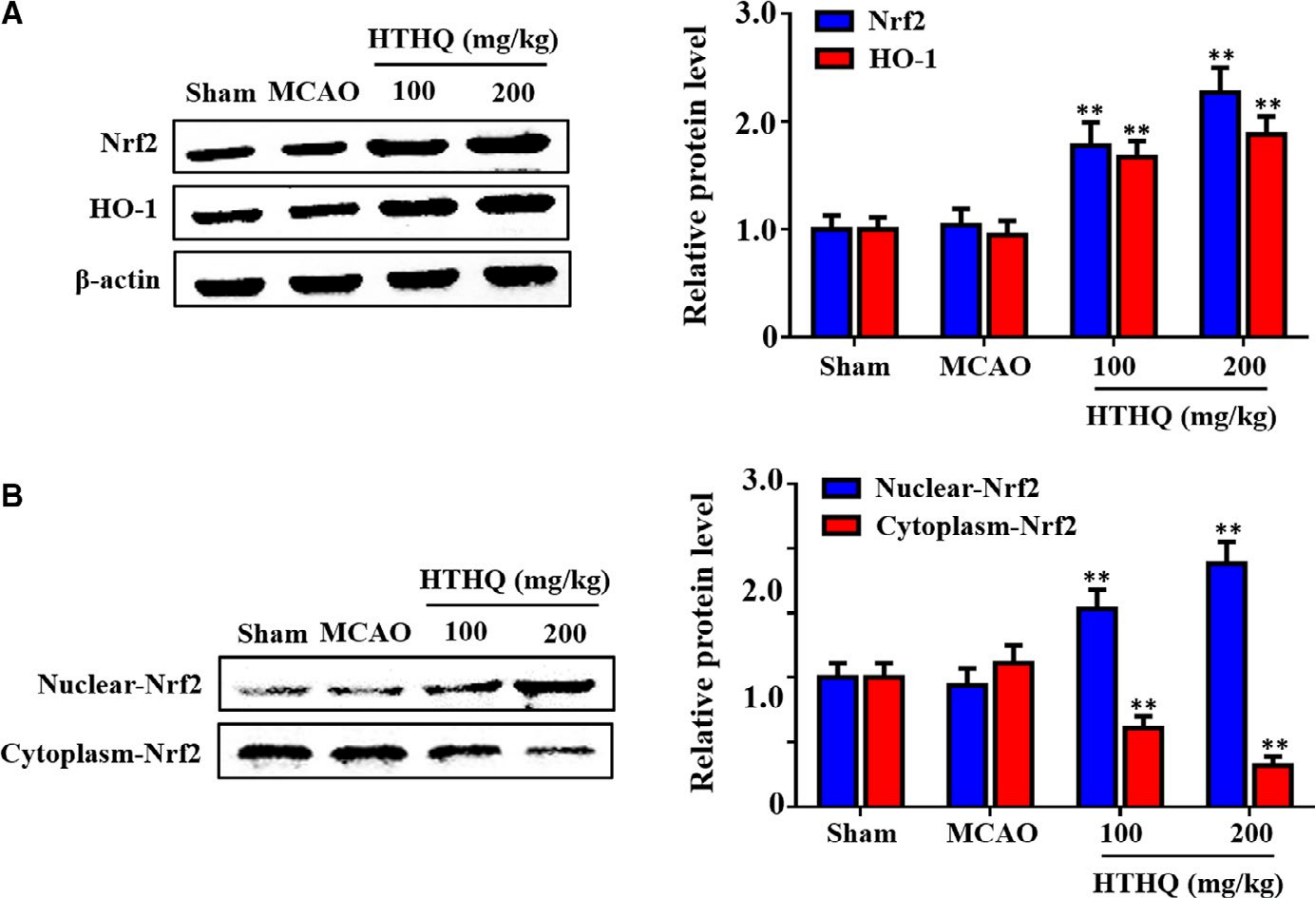
HTHQ against cerebral I/R injury via Nrf2 antioxidant pathway. A, Western blots showing the level of Nrf2 and HO‐1 in mice following cerebral I/R (n = 4). B, Western blots showing the level of nuclear Nrf2 and cytosolic Nrf2 in mice following cerebral I/R (n = 4). **P < .01 vs the MCAO group

Recent evidence has strongly suggested that the Nrf2 antioxidant pathway is critical regulators of cerebral I/R injury.^12^, ^13^ Thus, we investigated whether HTHQ protect against cerebral I/R injury is associated with Nrf2 antioxidant pathway. The results showed that the expression of Nrf2 and HO‐1 was also significantly increased after HTHQ treatment (Figure 4A). In addition, HTHQ treatment significantly increased significantly increased the levels of Nrf2 in the nucleus, while decreasing its expression in the cytoplasm compared with the MCAO group (Figure 4B). These findings indicate that HTHQ regulates cerebral I/R injury via Nrf2 antioxidant pathway.

### 3.5 HTHQ treatment attenuates oxidative stress in PC12 cells following H/R

**FIGURE 5:**
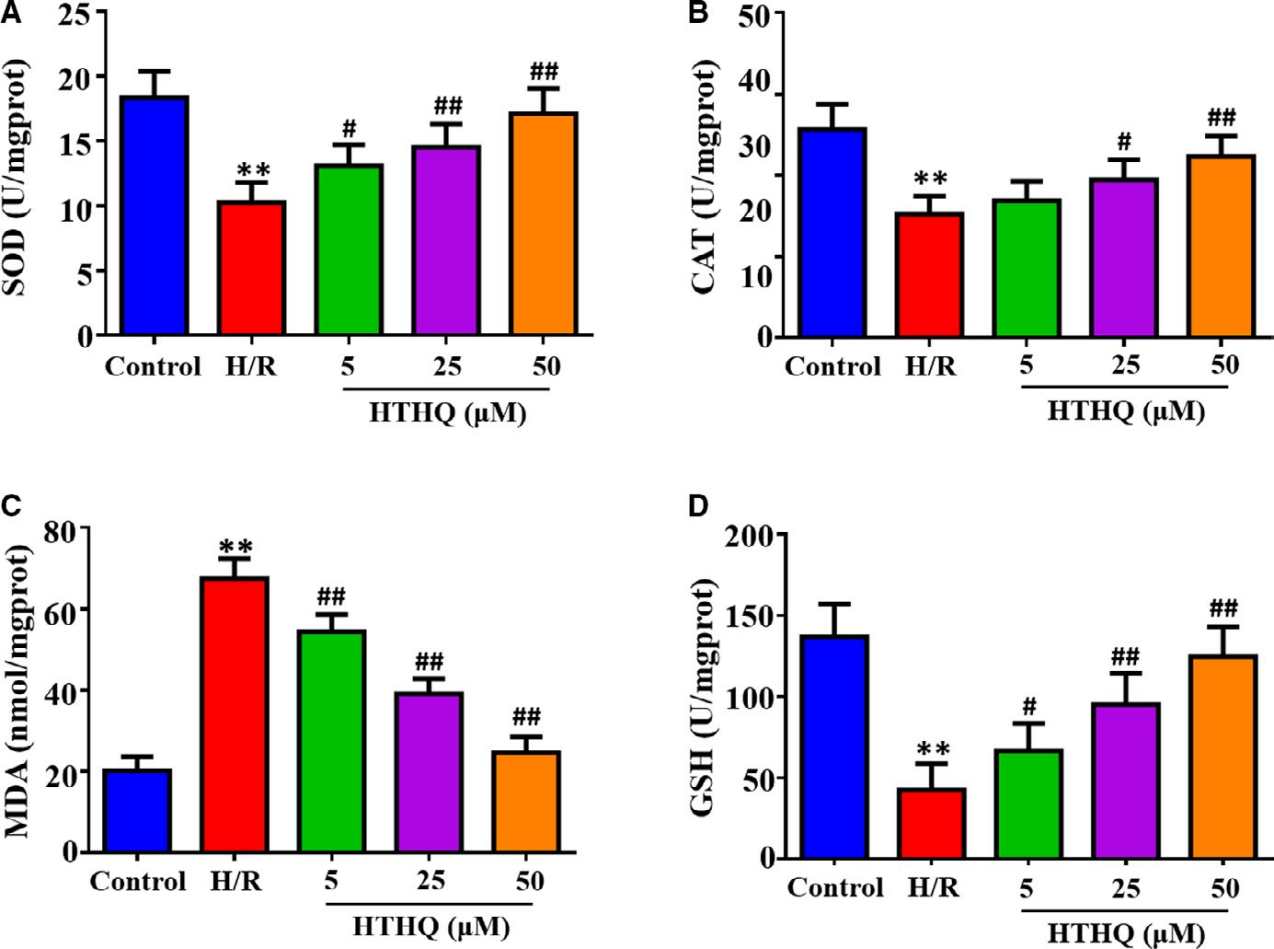
HTHQ treatment attenuates oxidative stress in PC12 cells following H/R. The levels of superoxide dismutase (SOD) (A), catalase (CAT) (B), malondialdehyde (MDA) (C) and glutathione (GSH) (D) in PC12 cells (n = 6). **P < .01 vs the control group; ^#^ *P* < .05 and ^##^ *P* < .01 vs the H/R group

To evaluate the cytotoxicity of HTHQ in PC12 cells, we performed a methyl tetrazolium (MTT) assay. We found that the viability of PC12 cells was not affected by treatment with HTHQ below 50 μmol/L (data not shown). Therefore, 50 μmol/L or less of the compounds was selected for subsequent research. To further assess the effects of HTHQ on oxidative stress following H/R, we measured the SOD, CAT, GSH activities and MDA contents in PC12 cells. The results revealed that the SOD, CAT and GSH activities in the H/R group were significantly lower and MDA contents were significantly higher than those in the control group (Figure 5A‐D). However, HTHQ treatment significantly restored SOD, CAT, GSH activities and decreased MDA contents compared with the H/R group (Figure 5A‐D). These findings indicate that HTHQ treatment attenuates oxidative stress following cerebral I/R.

### 3.6 HTHQ treatment decreases apoptosis of PC12 cells following H/R

**FIGURE 6:**
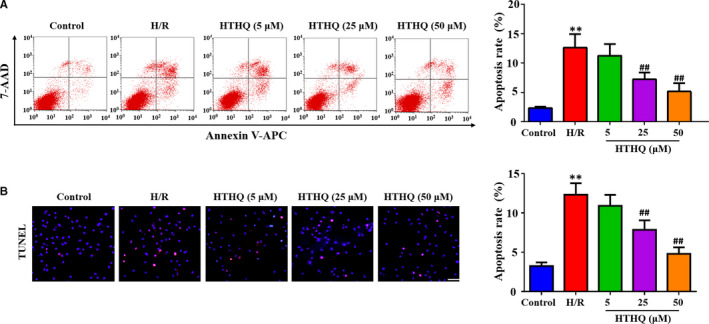
HTHQ treatment decreases apoptosis of PC12 cells following H/R. A, Flow cytometry analysis of cell apoptosis following H/R (n = 6). B, Images and quantifications of TUNEL‐positive cells following H/R (n = 5). **P < .01 vs the control group; ^##^ *P* < .01 vs the H/R group

To assess the neuroprotective effects of HTHQ, flow cytometry and TUNEL staining were used to measure the cell apoptosis. The results demonstrated that apoptotic index in the H/R group obviously increased compared with that in the control group, whereas HTHQ treatment significantly decreased neuronal cell death following H/R (Figure 6A,B).

### 3.7 The protection of HTHQ involves the Nrf2/HO‐1 pathway

**FIGURE 7:**
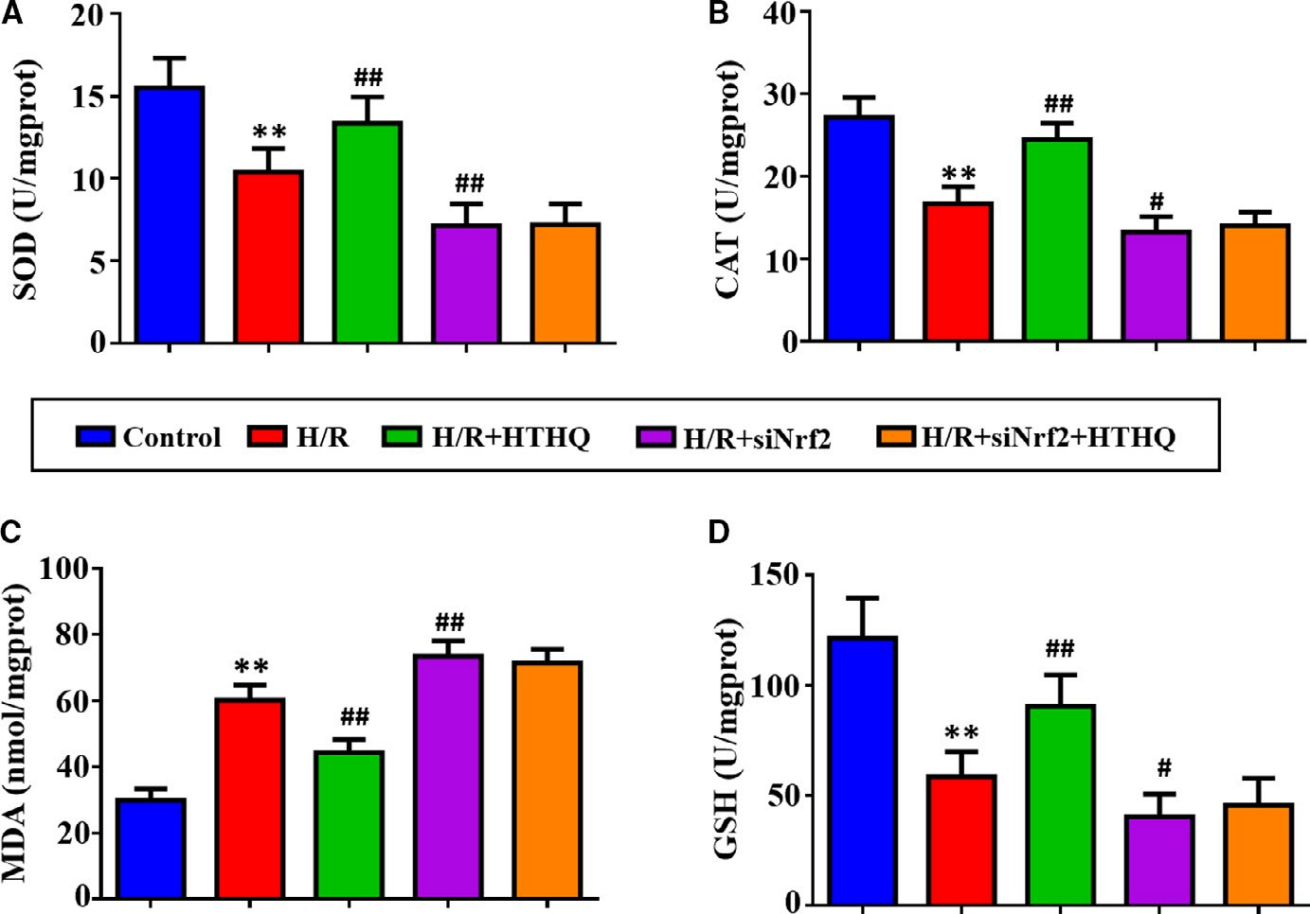
The antioxidant activity of HTHQ involves the Nrf2/HO‐1 Pathway. The levels of superoxide dismutase (SOD) (A), catalase (CAT) (B), malondialdehyde (MDA) (C) and glutathione (GSH) (D) in PC12 cells after si‐Nrf2 transfection (n = 6). **P < .01 vs the control group; ^#^ *P* < .05 and ^##^ *P* < .01 vs the H/R group

**FIGURE 8:**
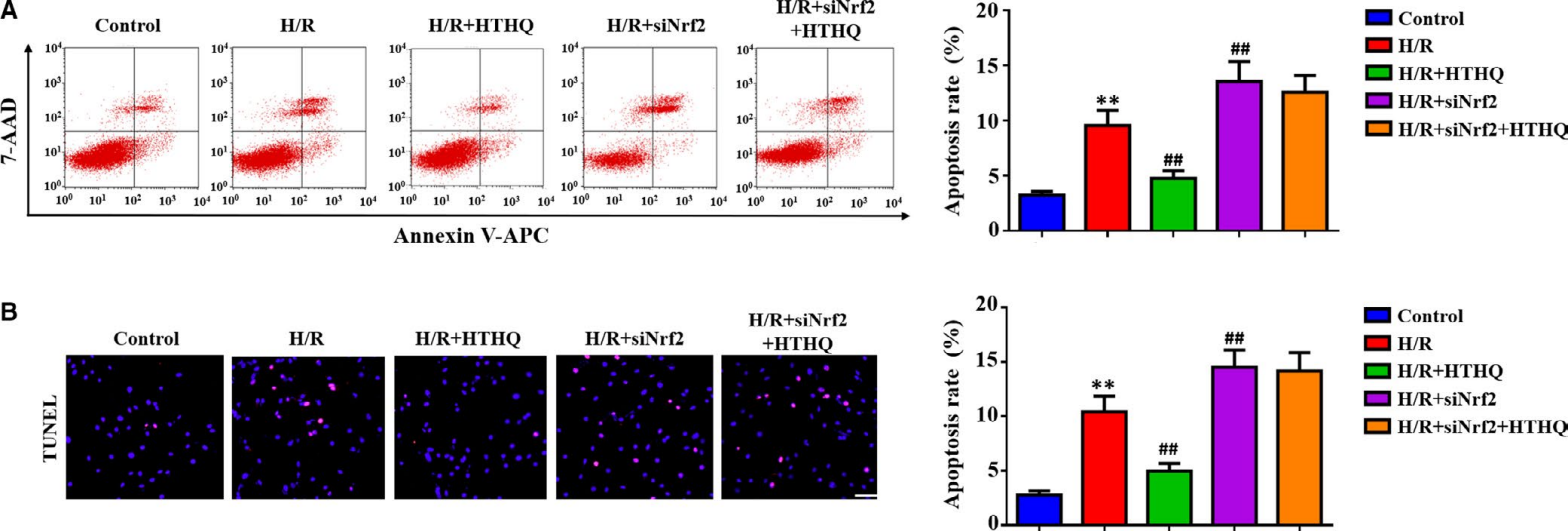
The anti‐apoptosis ability of HTHQ involves the Nrf2/HO‐1 Pathway. A, Flow cytometry analysis of cell apoptosis after si‐Nrf2 transfection (n = 6). B, Images and quantifications of TUNEL‐positive cells after si‐Nrf2 transfection (n = 5). **P < .01 vs the control group; ^##^ *P* < .01 vs the H/R group

To confirm the role of Nrf2/HO‐1 pathway in HTHQ‐mediated neuroprotection, we measure the oxidative stress and cell apoptosis after si‐Nrf2 transfection. The results showed that PC12 cells were more vulnerable to H/R‐induced oxidative stress after si‐Nrf2 transfection. Meanwhile, the HTHQ‐mediated protection was lost in PC12 cells transfected with siNrf2 (Figure 7A‐D). In consistent, knockdown of Nrf2 increases the apoptosis ratio after H/R, and HTHQ treatment did not affect neuronal apoptotic in PC12 cells transfected with siNrf2 (Figure 8A,B). Taken together, the neural protective effect of HTHQ is involved activation of Nrf2/HO‐1 pathway.

## 4 DISCUSSION

In this study, we demonstrated that HTHQ treatment ameliorated cerebral I/R injury in vivo, as demonstrated by the decreased infarct size, neurologic scores, oxidative stress and neuronal apoptosis. HTHQ treatment also increased the levels of Nrf2 and HO‐1. In addition, HTHQ treatment decreases oxidative stress and neuronal apoptosis of PC12 cells following H/R in vitro. Moreover, we provided evidence that PC12 cells were more vulnerable to H/R‐induced oxidative stress after si‐Nrf2 transfection, and the HTHQ‐mediated protection was lost in PC12 cells transfected with siNrf2. These data are noteworthy and suggest that the neural protective effect of HTHQ is Nrf2 dependent.

Stroke has become an important reason of death and permanent disability worldwide. A transient or permanent interrupt in blood supply frequently can lead to death of neurons in the brain and reperfusion of the blood supply may further aggravate injury in ischaemic brain tissue.^20^, ^21^ Many animal models have been executed to mimic human brain injury after stroke.^18^, ^22^, ^23^, ^24^ In this study, we employed the mice MCAO model and cellular H/R model to simulate cerebral I/R injury in vivo and in vitro. Infarct volume ratios, neurologic score and brain water content are the specific markers to estimate brain injury after stroke.^18^, ^23^ In this study, our results revealed that the infarct volume ratios, neurologic score and brain water content in the MCAO group obviously increased compared with that in the Sham group, whereas HTHQ treatment significantly decreased the infarct size, neurologic score and brain water content in a dose‐dependent manner.

Oxidative stress, the imbalance between ROS and antioxidant systems, has been proved to be an important pathological feature in cerebral I/R injury.^25^, ^26^ Under physiological conditions, ROS are generated during the metabolism of oxygen (O_2_). If excessive ROS accumulation or inactivation of the antioxidant defence systems, ROS will interact with proteins, lipids, ribonucleic acid and deoxyribonucleic acid, causing neuronal cell injury or apoptosis, and ultimately neuronal cell death.^27^, ^28^, ^29^ In response to cellular insults, enzymatic defence systems, such as SOD and CAT, and non‐enzymatic antioxidants GSH can inhibit ROS chain reactions and free radical formation.^30^, ^31^ Previous study demonstrated that the SOD, CAT and GSH activities were significantly lower and MDA contents were significantly higher in patients with acute ischaemic stroke or mice MCAO model.^32^, ^33^, ^34^

1‐O‐Hexyl‐2,3,5‐trimethylhydroquinone, one of the hydroquinone monoalkyl ethers, have an antioxidant and anti‐lipid peroxidative activity. The recent research indicates that HTHQ can protect dopaminergic neuronal cell against 3,4‐l‐Dihydroxyphenylalanine‐induced neurotoxicity by inhibiting ROS formation.^17^ Similarly, our results showed that HTHQ treatment significantly reduced the extent of oxidative stress by increasing cellular antioxidants SOD, CAT and GSH activity and decreasing MDA contents. The results indicated that HTHQ should have a certain scavenging ROS and suppressing lipid peroxidation effect and protect the brain against oxidative stress‐induced injury. Nevertheless, the precise mechanisms on how HTHQ exerts neuroprotection effect still unclear.

Nrf2, a master regulator of endogenous antioxidant defence, is an essential cellular system in place to counterbalance the production of ROS and protect cells against oxidative stress.^10^, ^35^ Studies showed that when cells are exposed to oxidative and electrophilic challenges, Nrf2 is activated, released from Kelch‐like ECH‐associated protein 1 (Keap1) and binds to the ARE sequences, then recruits the transcription of antioxidant defence genes in nuclear.^36^ In an experimental mouse model of MCAO‐induced cerebral I/R injury, knockout of Nrf2 significantly increased infarct size and neuronal apoptosis by exacerbating oxidative stress.^37^ Various small‐molecule activator, including sulforaphane, britanin, ursolic acid and flavonoids, have neuroprotective effects by up‐regulating the expression of Nrf2 and HO‐1 in models of cerebral I/R injury.^38^, ^39^, ^40^, ^41^ These researches further highlight that Nrf2 is a promising therapeutic target for the treatment of brain injury after stroke.

In the present study, we investigated the role of HTHQ treatment in Nrf2 signalling pathway. The results showed that HTHQ treatment significantly increased significantly increased the expression of Nrf2 in the nucleus, while decreasing its expression in the cytoplasm. As shown in our in vitro study, knockdown of Nrf2 increased the level of oxidative stress and apoptosis of PC 12 cells under H/R. In addition, the HTHQ treatment mediated neural protective effect was lost in PC12 cells transfected with siNrf2. HO‐1, which is regulated by Nrf2, has antioxidant, anti‐inflammatory and anti‐apoptotic activities by restoring redox homeostasis and reducing inflammation response.^42^, ^43^ HO‐1 has been reported to be important against brain injury after stroke, as demonstrated by increasing infarct size and neurological deficit in HO‐1 knockout mice.^44^ In this study, we also found that HTHQ treatment significantly increased HO‐1 expression in the brain after cerebral I/R injury. Taken together, we propose that the neuroprotective actions of HTHQ is related to an antioxidant and anti‐apoptotic effect and the mechanism is involving Nrf2/HO‐1 signalling pathway.

In conclusion, the present study indicates that HTHQ significantly ameliorates cerebral I/R injury in vivo and protect PC12 cells against H/R injury in vitro via Nrf2/HO‐1 pathway. These findings suggest that HTHQ could be a potential pharmacological agent for prevention or treatment of stroke.

## CONFLICT OF INTEREST

The authors declare that there is no conflict of interest regarding the publication of this article.

## AUTHOR CONTRIBUTION

**Chaoliang Tang:** Conceptualization (lead); Funding acquisition (lead); Investigation (lead); Project administration (lead); Resources (lead); Supervision (lead); Writing‐original draft (lead); Writing‐review & editing (lead). **Yida Hu:** Data curation (equal); Investigation (equal); Methodology (equal); Software (equal). **Haiyan Lyu:** Formal analysis (equal); Methodology (equal); Software (equal); Visualization (equal). **Jie Gao:** Methodology (equal); Writing‐review & editing (equal). **Jiazhen Jiang:** Data curation (equal); Investigation (equal); Software (equal); Writing‐review & editing (equal). **Xiude Qin:** Data curation (equal); Formal analysis (equal); Methodology (equal); Software (equal); Validation (equal). **Yuanbo Wu:** Data curation (equal); Formal analysis (equal); Funding acquisition (equal); Methodology (equal); Software (equal). **Jiawu Wang:** Conceptualization (equal); Formal analysis (equal); Methodology (equal); Software (equal). **Xiaoqing Chai:** Conceptualization (equal); Investigation (equal); Project administration (equal); Supervision (equal); Writing‐review & editing (equal).

## Data Availability

The data used to support the findings of this study are available from the corresponding author upon request.
